# CRISPR/Cas9‐enhanced ssDNA recombineering for *Pseudomonas putida*

**DOI:** 10.1111/1751-7915.13453

**Published:** 2019-06-25

**Authors:** Tomás Aparicio, Víctor de Lorenzo, Esteban Martínez‐García

**Affiliations:** ^1^ Systems and Synthetic Biology Program Centro Nacional de Biotecnología (CNB‐CSIC) Campus de Cantoblanco 28049 Madrid Spain

## Abstract

Implementation of single‐stranded DNA (ssDNA) recombineering in *Pseudomonas putida* has widened the range of genetic manipulations applicable to this biotechnologically relevant bacterium. Yet, the relatively low efficiency of the technology hampers identification of mutated clones lacking conspicuous phenotypes. Fortunately, the use of CRISPR/Cas9 as a device for counterselection of wild‐type sequences helps to overcome this limitation. Merging ssDNA recombineering with CRISPR/Cas9 thus enables a suite of genomic edits with a straightforward approach: a CRISPR plasmid provides the spacer DNA sequence that directs the Cas9 nuclease ribonucleoprotein complex to cleave the genome at the wild‐type sequences that have not undergone the change entered by the mutagenic ssDNA oligonucleotide(s). This protocol describes a complete workflow of the method optimized for *P. putida*, although it could in principle be applicable to many other pseudomonads. As an example, we show the deletion of the *edd* gene that encodes one key enzyme that operates the EDEMP cycle for glucose metabolism in *P. putida* EM42. By combining two incompatible CRISPR plasmids with different antibiotic selection markers, we show that the procedure can be cycled to implement consecutive deletions in the same strain, e.g. deletion of the *pyrF* gene following that of the *edd* mutant. This approach adds to the wealth of genetic technologies available for *P. putida* and strengthens its status as a chassis of choice for a suite of biotechnological applications.

## Introduction

A large number of techniques have become available in recent years that ease the editing of the genomes of different types on bacteria. In the case of *E. coli,* the development of the lambda Red technology (Datsenko and Wanner, 2000) supposed a great leap forward, not only providing advances in the knowledge of its physiology but in the development of several genome‐reduced strains (Kolisnychenko, 2002; Pósfai *et al*., 2006). A further development of this original approach involves the use of synthetic single‐stranded DNA (ssDNA) as the agent for introducing the changes at stake in the replication fork stimulated by the action of the β recombinase of the Red system (Ellis *et al*., 2001). Alas, the method has an inherent low level of efficiency, which makes identification of mutated clones difficult in the absence of selection (Ellis *et al*., 2001; Aparicio *et al*., 2016). This can be alleviated by either multiplexing the process (such as in the case of the so‐called multiplex automated genome engineering: MAGE; Wang *et al*., 2009) or by combining ssDNA recombineering with some type of counterselection. Since the Cas9 nuclease can be directed against a specific DNA by providing it with the desired sequence in the form of a CRISPR spacer, ssDNA recombineering can be combined with the nuclease for killing those clones that have not been modified (Jiang *et al*., 2013; Ronda *et al*., 2016). This approach is applicable not just to *E. coli*, but to other bacteria of biotechnological interest (Keasling, 2012; Calero and Nikel, 2019), including *Pseudomonas putida* (de Lorenzo, 2011; Martinez‐García and de Lorenzo, 2019). A large number of molecular tools and strategies have been developed over the years to implement a suite of modifications in the genome of this bacterium (Martínez‐García *et al*., 2014). This includes not only growingly efficient ways to enter changes through double‐homologous recombination process (Martinez‐Garcia and de Lorenzo, 2011, 2012; Wirth *et al*., 2019), but also the sort of merged ssDNA recombineering‐CRISPR/Cas9 just commented (Aparicio *et al*., 2016, 2017). An advantage of this technology is the possibility of selecting mutants with a reduced fitness that in the case of homologous recombination‐based methods would be outnumbered by wild‐type cells.

In this work, we report the formatting of the plasmid containing a CRISPR array following the Standard European Vector Architecture rules (Silva‐Rocha *et al*., 2013; Martinez‐Garcia *et al*., 2015). This new CRISPR plasmid was incorporated into the SEVA database (http://seva.cnb.csic.es/). On this basis, we describe a simple protocol to perform diverse types of genome modifications in *P. putida*. The procedure involves (i) ssDNA and spacer selection; (ii) cloning the spacer into the CRISPR plasmid; (iii) co‐transform the desired host with the ssDNA and the CRISPR plasmid bearing the spacer; (iv) confirm the deletion; and (v) cure the plasmids from the deleted strain. Moreover, we show the possibility of combining the use of two mutually exclusive CRISPR plasmids with different antibiotic selection markers for cycling a multi‐deletion process.

## Protocol

The procedure described in this paper consists of combining together two simple techniques (i) ssDNA recombineering to introduce the desired DNA change in the genome and (ii) CRISPR/Cas9 to efficiently counterselect the non‐modified bacterial clones to easily recover mutated clones with a non‐conspicuous phenotype. Here, we are going to illustrate this protocol with a 1 kb deletion example but it could be applied as well for DNA insertions, single nucleotide changes or big chunk deletions.

### Bacterial strains, media and chemicals

The *E. coli* bacterial strains used in this work are CC118 (Manoil and Beckwith, 1985) as the cloning host and HB101 (pRK600) as the helper strain for tri‐parental matings (Kessler *et al*., 1992). To perform the deletion experiments, we used the *Pseudomomas putida* KT2440 derivative named EM42 (Martinez‐Garcia *et al*., 2014). The list of plasmids used in this paper is described in Table 1. LB medium is used as the routine medium for growth of both *P*. *putida* and *E. coli*. In specific cases, we used the M9 minimal medium (Sambrook *et al*., 1989) supplemented with 0.2% (w/v) of either glucose or citrate as the sole carbon source. The use of citrate as C‐source is required for nutritional selection, such as in the case of matings to transfer plasmids from *E. coli* to *P. putida*. This C‐source allows to counterselect the *E*. *coli* donor and mating helper strains from the mating mix (Martinez‐Garcia *et al*., 2017). Moreover, when required media were supplemented with 50 μg ml^−1^ kanamycin (Km), 30 μg ml^−1^ chloramphenicol (Cm), 10 μg ml^−1^ or 15 μg ml^−1^ gentamycin (Gm) for *E. coli* or *P. putida*, respectively, 100 μg ml^−1^ streptomycin (Sm) for *P. putida* and 50 μg ml^−1^ for *E. coli*, and 20 μg ml^−1^ uracil. All oligonucleotides were purchased from Sigma‐Aldrich, dissolved in H_2_O to obtain either 5 μM oligo solutions for PCR and sequencing reactions or 100 μM stocks for ssDNA recombineering. The oligonucleotide stocks were stored at −20°C.

**Table 1:** Plasmids used in this work

Plasmid	Description and relevant characteristics	References
pSEVA658‐*ssr*	*xylS*‐Pm → *ssr*,* oriV* RSF1010; Gm^R^	Aparicio *et al*. (2017)
pSEVA421‐Cas9tr	Cas9 and tracrRNA; *oriV* RK2; Sm^R^/Sp^R^	Aparicio *et al*. (2017)
pSEVA2316	SEVA CRISPR array; *oriV* pBBR1; Km^R^	This work
pSEVA5316	SEVA CRISPR array; *oriV* pBBR1; Tet^R^	This work
pSEVA2316‐edd1	pSEVA2316 derivative bearing the edd1 spacer	This work
pSEVA231‐C‐edd1	pSEVA231‐CRISPR derivative bearing the edd1 spacer	This work
pSEVA231‐C‐edd3	pSEVA231‐CRISPR derivative bearing the edd3 spacer	This work
pSEVA5316‐pyrF1	pSEVA5316 derivative bearing the pyrF1 spacer	This work
pSEVA231‐CRISPR	CRISPR array; *oriV* pBBR1; Km^R^	Aparicio *et al*. (2017)
pSEVA231	MCS; *oriV* pBBR1; Km^R^	Silva‐Rocha *et al*. (2013)
pSEVA531	MCS; *oriV* pBBR1; Tet^R^	Silva‐Rocha *et al*. (2013)
pRK600	Mating helper plasmid; *oriV* ColE1, RK2(*mob* ^*+*^ *tra* ^*+*^); Cm^R^	Kessler *et al*. (1992)

Cm, chloramphenicol; Gm, gentamycin; Km, kanamycin; MCS, multiple cloning site; Sm: streptomycin; Sp: spectinomycin; Tet: tetracycline.

### Construction of a bacterial strain harbouring plasmids with an inducible recombinase and Cas9

The first thing we need to start a deletion project in our selected host is to introduce two different plasmids that are required for the technique. One is the pSEVA658‐*ssr* plasmid that expresses the Ssr recombinase in an inducible way (Aparicio *et al*., 2016); and the second one is the pSEVA421‐Cas9tr that constitutively expresses the Cas9 nuclease and the tracrRNA (Aparicio *et al*., 2017). We recommend to do it serially, introducing one plasmid first and then the other. We tested both possible order combinations, first introducing the Ssr‐containing plasmid and then the Cas9 vector; and the other way around, the Cas9 plasmid first and then the Ssr vector. Both permutations worked fine in different *P. putida* strains. To transform these plasmids, even though more time‐consuming, we recommend the conjugation option as the choice method not only to increase the efficiency but also to maximize the correct integrity of these plasmids. For that reason, we describe below the mating protocol, and in the section dealing with the interference test, we will explain the electroporation procedure.

### Introduction of plasmids in the strain of choice

The protocol described here is a simplified version of Martinez‐Garcia *et al*. (2017) without measuring OD_600_ of cultures and not using filters to lay the bacteria on.

- From the −80°C frozen stocks grow aerobically overnight liquid cultures of:
  - *E. coli* CC118 donor cells harbouring the plasmid to be transferred into *P. putida* (pSEVA658‐*ssr* or pSEVA421‐Cas9tr) in 2 ml LB with the appropriate antibiotic (Gm or Sm) at 37°C.
  - *E. coli* HB101 helper strain (Boyer and Roulland‐Dussoix, 1969), that encodes the transfer and mobilization functions in the plasmid pRK600, in 2 ml LB with Cm at 37°C.
  - *P. putida* recipient strain in 2 ml LB at 30°C.
- Take 800 μl of the grown cultures, transfer them to a 1.5 ml Eppendorf tube and centrifuge at 9300 *g* for 2 min. Discard the supernatant and add 800 μl of 10 mM MgSO_4_. Then, suspend the pellet gently.
- Centrifuge at 9300 *g* for 2 min. Discard the supernatant, add 800 μl of 10 mM MgSO_4_ and suspend the pipetting up and down.
- Transfer 100 μl of each of the three bacterial strains to a new 1.5 ml Eppendorf tube. Centrifuge at 9300 *g* for 2 min. Discard the supernatant and add 20 μl of 10 mM MgSO_4_.
- Spot the 20 μl mating mixture onto a dried and prewarmed LB agar plate. Let it dry for 5 min at room temperature and then incubate the LB agar plate at 30°C for 6 h in an upward position.
- Using a bended yellow tip scrape the mating spot and suspend it in 1 ml of 10 mM MgSO_4_.
- Plate different dilutions (normally, 10^−3^, 10^−2^ and 10^−1^) onto M9 minimal medium with 0.2% (w/v) citrate supplemented with the appropriate antibiotic (15 μg ml^−1^ Gm for pSEVA658‐*ssr* or 100 μg ml^−1^ Sm for pSEVA421‐Cas9tr). Incubate overnight at 30°C.
- Select a few colonies and re‐streak them into M9 + 0.2% (w/v) citrate+ antibiotic and check the presence of the correspondent plasmid by miniprep and restriction.
- Prepare a frozen stock of the correct strain in LB 20% (v/v) glycerol and store at −80°C.
- Repeat the whole process to introduce the second plasmid.

### Cloning the spacer into the CRISPR plasmid

This section explains how to design and anneal the appropriate spacers for their cloning into the empty CRISPR plasmids.

### Construction of a SEVA CRISPR plasmid

**Figure 1:**
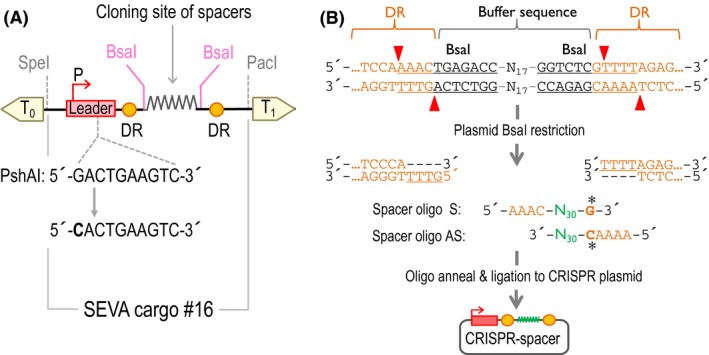
Details of the relevant parts of the CRISPR plasmid. A. Schematic representation of the novel SEVA cargo for CRISPR. This new cargo contains the elements for the CRISPR array and has been designated with the code #16. The T_0_ and T_1_ transcriptional terminators flanking the cargo region are represented as arrows that indicate the direction of termination. The leader region of the CRISPR array (represented in red within the cargo module) is a 132 bp AT‐rich sequence that contains the promoter (a red arrow in the figure) that is responsible for the transcription of the crRNA. A detail of the PshAI restriction site in the original sequence is shown together with the modified version, lacking the restriction site, in the new CRISPR plasmid. The yellow dots represent the 36 bp direct repeats (DR) that flank the spacer. The two BsaI sites indicate the place designated to clone the appropriate spacer DNA. B. Closer look at the DNA details of the business region of the CRISPR array. The CRISPR array DNA sequence of pSEVA2316 derives from pSEVA231‐CRISPR (Aparicio *et al*., 2017), and this one in turn comes from the original pCRISPR (Jiang *et al*., 2013). BsaI restriction sites are underlined, and the red arrows indicate the specific DNA positions where the enzyme cuts the DNA. DRs are depicted in orange. Details of the DNA sticky ends generated in a CRISPR plasmid digested with BsaI and the sequence necessary to incorporate a piece of DNA into this site are also shown. The asterisk denotes the specific nucleotide that has to be included into the oligonucleotides to reconstruct the direct repeat (DR) in the CRISPR array after the ligation of both fragments. N_30_, in green, stands for the spacer sequence.

Even though the pSEVA231‐CRISPR plasmid was proven to be fully functional as stated by Aparicio *et al*. (2017), it does not match the SEVA standardization rules (Silva‐Rocha *et al*., 2013; Martinez‐Garcia *et al*., 2015). This is because of the presence of a PshAI restriction site in the natural sequence of the leader region of the CRISPR array. Together, both PshAI and SwaI are two key enzymes for the SEVA standard since they are required to swap the antibiotic resistance marker in those plasmids. For that reason, we decided to apply the SEVA format (Silva‐Rocha *et al*., 2013; Martinez‐Garcia *et al*., 2015) to the CRISPR module as a new cargo for the collection and test its functionality. To do that, we needed to eliminate PshAI restriction site present in the promoter region of the CRISPR element. So, we changed the natural sequence present in the pSEVA231‐CRISPR plasmid 5′‐GACTGAAGTC‐3′ for the newly designed 5′‐**C**ACTGAAGTC‐3′. On this basis, we outsourced the complete synthesis of the 395 bp CRISPR DNA module to GeneCust. Since the resulting synthesized DNA was cloned in the pUC57 vector, the module was excised with the flanking enzymes EcoRI and BamHI and cloned into those sites in the cargo region of pSEVA231 and pSEVA531 plasmids. This new module was assigned with the SEVA cargo code #16 (Fig. 1A) and the resulting plasmids named pSEVA2316 and pSEVA5316.

### CRISPR plasmid extraction

This protocol is used here to prepare the CRISPR plasmid (pSEVA2316, pSEVA5316 or pSEVA231‐CRISPR; Table 1) that is going to be the receptor of the spacers but it also works to extract the plasmids containing the spacers for the electroporation required at the final steps of the deletion procedure. For plasmid extraction, we normally use the QIAprep Spin Miniprep^TM^ Kit (Qiagen Inc., Valencia, CA, USA) and follow the manufacturer's instructions.

- From the frozen stock, inoculate with the *E. coli* strain that harbours the pSEVA2316 (or any other of the CRISPR plasmid) in a 100 ml flask containing 20 ml of LB plus the appropriate antibiotic and grow it aerobically at 37°C overnight.
- Transfer the culture to a 50 ml Falcon tube and centrifuge the whole culture at 3220 *g* for 20 min at room temperature.
- Discard the supernatant and proceed with the plasmid extraction adding the volume recommended for 4 reactions of buffer 1 (in the particular case of Qiagen, we add 1000 μl of buffer 1).
- Vortex to re‐suspend the pellet and distribute the total 1 ml into four Eppendorfs containing 250 μl each.
- Proceed as indicated in the instructions of the plasmid extraction kit provider.
- Elute the plasmid DNA by adding 100 μl of H_2_O to each tube. Then, concentrate the DNA in a SpeedVac for 30 min and finally mix the liquid of the four Eppendorfs into one tube.
- Quantify the plasmid DNA concentration with a NanoVue Plus (GE Healthcare, Chicago, IL, USA).

### Restriction of the CRISPR plasmid with BsaI and backbone purification

**Figure 2:**
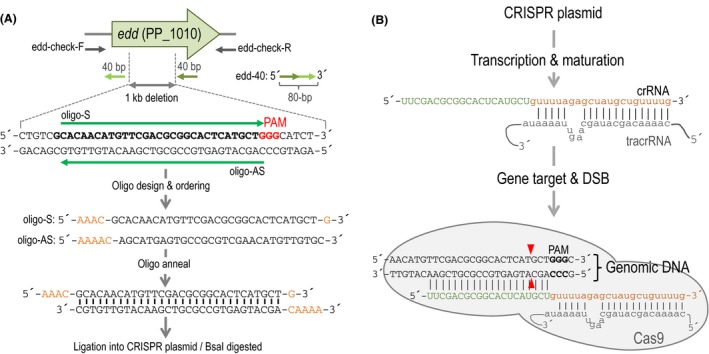
General diagram of the spacer design process with a scheme representing the production of an active crRNA targeting the selected gene by Cas9. A. Outline of the first steps of the deletion protocol. Special details have been put to the oligonucleotide selection to clone a spacer. First, one needs to identify a potential PAM sequence (5′‐NGG‐3′) and select the 30 bp adjacent. In orange are depicted the nucleotides that have to be added to the oligos to create overhangs for cloning on the BsaI site. Finally, order the sense (S) and the antisense (AS) oligonucleotides to your favourite company. Also, the ssDNA (edd‐40) is depicted in the figure as green arrows: two arms of 40 bp of homology flanking the area to be deleted which accounts to a total of 80 bp of the single oligonucleotide. B. Diagram of the crRNA formation and assembly on the ribonucleoprotein Cas9 complex. The upper part shows the alignment of the crRNA and tracrRNA molecules. Within the crRNA, the spacer part is depicted in green and the DR in orange, while the tracrRNA is shown in grey. To simplify the view, only the complementary part of the tracrRNA is depicted in the representation. The lower part sketches the Cas9:crRNA:tracrRNA complex sitting on its genomic target sequence. The red arrows indicate the position of the breaks introduced by Cas9 within the target gene.

The empty CRISPR plasmid has to be digested with the restriction enzyme BsaI or BsaI‐HF (NEB, Ipswich, MA, USA) to clone the designed spacers (Figs 1B and 2A).

Prepare the following restriction mix:

- Add 10 μl of the 10× CutSmart buffer.
- Add 10 μl of the 10× bovine serum albumin (BSA).
- Complete with 78 μl of plasmid DNA
- 2 μl of BsaI
- Incubate for 2 h at 37°C.
- Add 17 μl of 6× Gel loading Dye (NEB)., Ipswich, MA, USA

Once the plasmid is digested, proceed to purify the plasmid backbone DNA from an agarose gel. This ensures the elimination of the DNA buffer sequence from the restriction mixture.

- Prepare a 1% (w/v) agarose gel and load the restriction sample.
- Purify the 3.3 kb linear fragment of the pSEVA2316 plasmid (or 3.7 kb for pSEVA5316) using an appropriate DNA extraction kit. We normally use the NucleoSpin^™^ Gel and PCR clean‐up kit (Macherey‐Nagel GmbH & Co. KG, Düren, Germany).
- Quantify the purified plasmid DNA concentration spectrophotometrically. Also, visualize the DNA by inspection on a 1% (w/v) agarose gel. Purified plasmid DNA can be kept at −20°C until further use.

### Spacer selection for crRNA

In order to use the CRISPR/Cas9 system as a counterselection method, we first need to clone a proper spacer into the BsaI sites of the plasmid that contains the CRISPR array (pSEVA2316, pSEVA5316 or pSEVA231‐CRISPR; Table 1). The cloned spacer is transcribed and processed into a proper crRNA that together with the tracrRNA guides the Cas9 nuclease to the target position in the chromosome (Gasiunas *et al*., 2012; Jinek *et al*., 2012) (Fig. 2B). Once the Cas9:tracrRNA:crRNA complex finds its genomic target and it is adjacent to a PAM, the nuclease introduces double‐strand breaks (DSB) that are lethal if not repaired. For that reason, the spacer sequence has to be contained within the region that is intended to be deleted or substituted in order to allow mutant clones to escape the scan of the Cas9 nuclease. In the case of a gene/operon deletion, the spacer can be located anywhere within the eliminated DNA. When intending to perform single base substitutions, it is crucial that the distance of the modified nucleotide to the PAM is no more than 3‐nt away (Aparicio *et al*., 2017). This requirement limits the possibility of single base substitutions to the proximity of PAM sequences in that area. Also, it is important that the mismatch between the genome and mutagenic oligo is loosely recognized by the endogenous MMR system, otherwise it will be automatically repaired (Aparicio *et al*., 2017). The selection of spacer sequences could be done manually or by the use of a specific online software tool such as CRISPOR (http://crispor.org) (Haeussler *et al*., 2016) and CRISPy‐web (https://crispy.secondarymetabolites.org/#/input; Blin *et al*., 2016). However, in this section we describe the manual procedure applied to the deletion of the *edd* gene of *P. putida* EM42. We strongly recommend to select at least two or three spacers and test them at the same time to ensure that at least one of them works properly. Even consuming more resources, this approach could save time. Then, use the spacer that shows the best efficiency in the interference test (see below).

- Start by scanning your target gene or region of interest to identify different protospacer‐adjacent motifs (PAM: 5′‐NGG‐3′) in any of the DNA strands.
- Then, select the 30 nucleotides immediately adjacent to the PAM in the 5′direction, such as 5′‐N_30_‐NGG‐3′ (where N could be any of the four nucleotides) and order the 30‐nucleotide sense oligo (S), importantly without including the PAM in its sequence, and the corresponding antisense oligo (AS) (Fig. 2A). We use 30 bp spacers since this approach uses a dual RNA‐guided system that contains the CRISPR array in one plasmid and the tracrRNA together with the Cas9 nuclease in another (Jiang *et al*., 2013). Once the pre‐crRNA is transcribed, it hybridizes with the tracrRNA and is processed into a matured and shorter crRNA (Deltcheva *et al*., 2011; Jinek *et al*., 2012). In order to allow the cloning of the spacer into the BsaI sites of the CRISPR plasmid, we add the corresponding nucleotides to both ends of the S and AS oligo to create the proper overhang sequences to anneal with the BsaI‐digested plasmid. To do that, add the following AAAC overhang nucleotides to the 5′‐end and a G to the 3′‐end of the S oligo. Respectively, add the following AAAAC bases to the 5′‐end of the AS oligo. The G added to the 3′‐end of the S primer and the last C of the nucleotides included to the 5′‐end of the AS oligonucleotide do not form part of the BsaI sticky end *per se* but allows to properly reconstruct the downstream direct repeat of the CRISPR array after the ligation of the spacer. A schema of this process is indicated in Figs 1B and 2A.
- The S and AS oligonucleotides are usually ordered with 5′‐phosphorylation. However, oligonucleotides can be ordered without phosphorylation since the BsaI‐digested plasmid backbone will contribute with the 5′‐phosphate end needed for DNA ligation. Even though this would produce a circular molecule with two nicks, it still transforms efficiently (Sambrook *et al*., 1989). We have tried both conditions and although the phosphorylated option works slightly better than the non‐phosphorylated one, this last choice is cheaper and oligonucleotides are delivered faster.

### Annealing of spacer oligonucleotides

- Add the required volume of H_2_O to the lyophilized spacer oligonucleotides to obtain a concentration of 100 μM. Vortex both tubes for 30 s and incubate them at RT for 5 min to dissolve them.
- Prepare the annealing mix by adding 45.5 μl of H_2_O, 2.5 μl of 1.0 M NaCl, 1 μl of oligo S (100 μM) and 1 μl of the oligo AS (100 μM) into a PCR tube.
- Place the PCR tube with the mix in a thermocycler with the following annealing programme: 5 min at 95°C; then, 1 min at 95°C and ramp down 1°C per cycle for 72 cycles and end by keeping the temperature at 10°C.
- Take 10 μl of the annealed S:AS oligonucleotides and dilute it with 90 μl of H_2_O to obtain a 0.2 μM concentration. The annealed oligonucleotides stocks can be stored at −20°C for future use.

### Cloning of spacers into the CRISPR plasmid

In the text below, pSEVA2316 (Km^R^) is used as example but the same procedure applies to any other CRISPR plasmid of choice. If using a different CRISPR plasmid, change accordingly the antibiotic used for selection during the protocols of cloning, interference and recombineering‐CRISPR/Cas9. To start this process, thaw the BsaI‐restricted pSEVA2316 plasmid and the diluted annealed oligonucleotides and prepare the following ligation mixture:

- Add 10 μl of the 2× quick ligation buffer
- Include 6 μl of H_2_O
- Add 50 to 100 ng of the linearized pSEVA2316.
- Add 1 μl of the diluted annealed oligonucleotides.
- 1 μl of quick ligase
- Incubate 5 min at room temperature

After the ligation process, we can directly proceed with the transformation step. To do that, we must have already prepared chemically or electrocompetent *E. coli* cells.

- Take 10 μl of the ligation mixture and add to competent cells of your favourite laboratory strain of *E. coli* (we normally use chemically competent cells of *E. coli* CC118).
- Incubate 15 min in ice.
- 1 min and 30 s at 42°C.
- Place for 5 min in ice.
- Add 900 μl of LB to the 100 μl of competent cells and incubate for 1 h at 37°C aerobically.
- Centrifuge at 7200 *g* for 2 min; discard 900 μl of the supernatant and use the 100 μl leftover to suspend the pellet. Then, plate everything onto an LB agar plate plus 50 μg ml^−1^ of Km. Incubate overnight at 37°C.
- Re‐streak a few clones to a fresh LB agar plate supplemented with Km to isolate individual clones.
- Extract plasmids from re‐streaked clones and send to sequence with either oligonucleotide PS1 or PS2 (Table 2) to verify the presence of the spacer.
  **Table 2:** Oligonucleotides used in this work

Name	Sequence 5′→3′	Usage	References
PS1	AGGGCGGCGGATTTGTCC	To sequence the cargo region of pSEVA plasmids	Silva‐Rocha *et al*. (2013)
PS2	GCGGCAACCGAGCGTTC	To sequence the cargo region of pSEVA plasmids	Silva‐Rocha *et al*. (2013)
PS3	GAACGCTCGGTTGCCGC	To sequence the gadget and selection marker of pSEVA plasmids	Silva‐Rocha *et al*. (2013)
edd‐40	*GCCCTGGAAGCGCACCACCGCGACAAAGTCACGCTCCAGC*TCACTTCACGGCTGGCGATCGCCAGCTCCATGCCCGGCTC	Mutagenic oligo to delete a 1 kb fragment of the *edd* gene	This work
cr‐edd‐1‐S	AAACGCACAACATGTTCGACGCGGCACTCATGCTG	Oligonucleotide to obtain the *edd*‐1 spacer	This work
cr‐edd‐1‐AS	AAAACAGCATGAGTGCCGCGTCGAACATGTTGTGC	Oligonucleotide to obtain the *edd*‐1 spacer	This work
cr‐edd‐3‐S	AAACCCGCAGCCTGGGCGATCGCCGGGATGTGCAG	Oligonucleotide to obtain the *edd*‐3 spacer	This work
cr‐edd‐3‐AS	AAAACTGCACATCCCGGCGATCGCCCAGGCTGCGG	Oligonucleotide to obtain the *edd*‐3 spacer	This work
edd‐check‐F	TAAACCGCCCTTACAATTAG	Diagnose deletion of *edd*	This work
edd‐check‐R	ACCAACGCAACCTTGTAG	Diagnose deletion of *edd*	This work
pyrF‐B‐np	*ACAGGCATCGGTGGTTCGGCACAGGCCCTTGCTGGACAGCCGCAGGTTAA*GGGCAGGGTCTCTTGGCAAGTCGAAAACGGCGCGCATTGTAAACGAAGTG	Mutagenic oligo to delete the complete *pyrF* gene	Aparicio, *et al*. (2019)
cr‐pyrF‐1‐S	AAACTTCGGGCATTGTCGAAACCCTGTGTGACAAG	Oligonucleotide to obtain the *pyrF* spacer	Aparicio *et al*. (2017)
cr‐pyrF‐1‐AS	AAAACTTGTCACACAGGGTTTCGACAATGCCCGAA	Oligonucleotide to obtain the *pyrF* spacer	Aparicio *et al*. (2017)
PYRF‐F	CGAGGGCTATGATGAGTATC	Diagnose deletion of *pyrF*	Aparicio *et al*. (2016)
PYRF‐R	GTCAGGTGAAGAGCAAAGAG	Diagnose deletion of *pyrF*	Aparicio *et al*. (2016)
- Select a correct clone and re‐streak in a fresh LB+Km agar plate and incubate it at 37°C overnight. Then, prepare a frozen stock by adding 2.5 ml of LB 20% (w/v) glycerol to the agar plate and with the help of a bended yellow tip scrape all cells, transfer the supernatant to cryovial and store it at −80°C.

### Interference test: checking the efficiency of spacers

**Figure 3:**
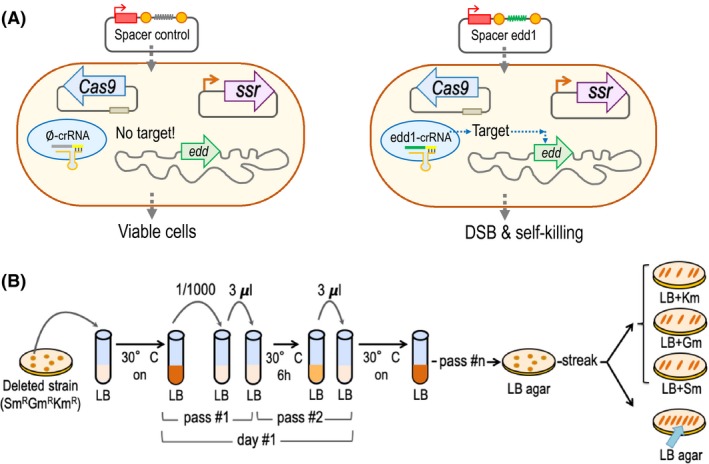
Interference experiment overview and plasmid curing. A. General outline of the interference experiment to test the efficiency of the selected spacers. B. Step‐by‐step guide to successfully cure the three plasmids after the deletion experiment. Once the deleted strain is obtained, inoculate it in liquid LB without antibiotics and perform several passes as indicated in the figure, plating the culture in LB solid media at the end of the process. Re‐streak colonies on LB plates containing each antibiotic to localize clones sensitive to all antibiotics (illustrated by a blue arrow in the picture).

Not all spacer sequences have the same efficiency guiding the Cas9 nuclease and it is not well understood what determines that. The aim of this optional experiment was to test the efficiency of the selected spacers to guide the Cas9 to target the chromosome and kill the cell. This procedure will allow us to select the most efficient spacer in a fast, easy way. Having one spacer that works efficiently ensures the successful use of CRISPR/Cas9 as a counterselection technique in a ssDNA recombineering experiment. Briefly, *P. putida* EM42 previously transformed with the plasmids pSEVA658‐*ssr* and pSEVA421‐Cas9tr, as described before, is electroporated in parallel with the CRISPR plasmids containing the spacer and also the control plasmid (pSEVA231‐CRISPR or pSEVA2316). Cells transformed with the control plasmid should produce a Cas9 complex without an effective target within the *P. putida* genome, rendering viable transformant clones (Fig. 3A). When using a different organism, it is important to ensure that the buffer sequence of the control plasmid has no match in that bacterium as previously checked *for P. putida* KT2440 (Aparicio *et al*., 2017). Nevertheless, in the case of cells transformed with the plasmids harbouring the designed spacers, the Cas9 complex will be able to identify the target adjacent to a PAM motif, introducing double‐strand breaks (DSB) in the genome that lead to bacterial death (Fig. 3A). To perform the interference experiment, proceed as follows:

- Start by purifying and quantifying the control plasmid (pSEVA2316 or pSEVA231‐CRISPR) and the CRISPR vectors harbouring the appropriate spacers (pSEVA231‐C‐edd1, pSEVA231‐C‐edd3 and pSEVA2316‐edd1; Table 1). To do that, follow the procedure described in the CRISPR plasmid extraction section.
- Inoculate a *P. putida* strain that harbours the pSEVA658‐*ssr* and pSEVA421‐Cas9tr in a 100 ml flask containing 20 ml of LB supplemented with Gm and Sm. Grow that culture aerobically overnight at 30°C.
- Collect the culture in a 50 ml Falcon tube and proceed to prepare electrocompetent cells.
- Centrifuge at 3200 *g* for 10 min at room temperature (RT) and discard supernatant.
- Add 10 ml of 300 mM sucrose and gently mix to suspend the cellular pellet.
- Centrifuge at 9300 *g* for 2 min at RT. Discard supernatant.
- Add 1 mL of 300 mM sucrose and gently mix and transfer the supernatant to 2 ml Eppendorf tube.
- Centrifuge at 9300 *g* for 2 min at RT. Discard supernatant gently (bacterial pellet could be loosely attached to the tube), add 800 μl of 300 mM sucrose and re‐suspend cells.
- Repeat step viii at least three times.
- Finally, add 400 μl of 300 mM sucrose (during this process cells are washed and concentrated 50‐fold).
- Add 100 ng of each plasmid directly to the side of one of the walls of a 2‐mm gap width electroporation cuvette.
- Transfer 100 μl aliquots of competent cells into each electroporation cuvette. We need one aliquot for the control plasmid (pSEVA2316) and one aliquot per spacer to be tested.
- Electroporate at 2.5 kV and quickly add 900 μl of LB supplemented with Sm and Gm.
- Let electroporated cells to recover for 2 h at 30°C with shaking.
- To enumerate viable cells, plate appropriate dilutions (10^−6^ and 10^−7^) into LB agar plus Sm+Gm. Then, to count transformant clones plate dilutions (10^−2^, 10^−1^ and 10^−0^) into LB agar plus Sm+Gm+Km.
- Incubate plates at 30°C until colonies appear and count CFUs. By the naked eye, there should be a clear difference of CFUs of transformant clones between the control and spacer plasmids while numbers of viable cells should be equal. To calculate the transformation efficiency, divide the CFUs on LB+Sm+Gm+Km (recombinant clones) by the CFUs on LB+Sm+Gm (viable cells) and normalize that to 10^9^ cells. Then, calculate the ratio of transformation efficiencies of the control plasmid versus the spacer ones. This number should be close to 100 to ensure an efficient counterselection.

## ssDNA recombineering with CRISPR/Cas9 counterselection

### Oligonucleotide design for ssDNA recombineering

Upon selection of a target gene, one have to start by designing a proper mutagenic oligonucleotide to perform the desired deletion experiment. The total length of the ssDNA should be around 80 or 90 bp, containing around 40 nucleotides of upstream and another 40 bp of downstream homology in the regions flanking the area to be deleted (Fig 2A). In the case of single substitutions, the mutation should be included in the middle part of the recombinogenic oligonucleotide and is important to take into account the effect of the mismatch repair system (MMR) of the bacterial host (Babic *et al*., 1996; Aparicio *et al*., 2017). Then, it is recommended to design the mutagenic oligo against the lagging strand (Ellis *et al*., 2001). This requires to know, *a priori*, the genomic coordinates of the *oriC* and *dif* regions in the organism of choice to be able to define the two replichores, positioning the leading/lagging strand in each one (Carnoy and Roten, 2009). In the case of not knowing those features, just design oligonucleotides for the two strands and test them both. The last aspect to consider is to minimize as much as possible the folding energy of the mutagenic oligo (preferentially ∆G > −12.5 kcal mol^−1^ for *E. coli* and > −16 kcal mol^−1^ for *P. putida*). For more details, see Aparicio *et al*. (2016).

### Induction of the Ssr recombinase

- Inoculate a 100 ml flask containing 20 ml of LB supplemented with 15 μg ml^−1^ Gm and 100 μg ml^−1^ Sm directly from the −80°C frozen stock of *P. putida* containing pSEVA658‐*ssr* and pSEVA421‐Cas9tr and incubate at 30°C aerobically overnight.
- Induce the expression of the Ssr recombinase by adding 1 mM 3‐methylbenzoate (3 MB) that activates the transcription driven by the XylS‐P_*m*_ system.
- Incubate the induced culture for 3 h at 30°C aerobically.
- After that time, collect the whole bacterial culture and proceed to prepare electrocompetent cells as described before.

### ssDNA and CRISPR plasmid electroporation

- Prepare a 100 μM stock solution of the ssDNA mutagenic oligo.
- Add 1 μl of the ~100 μM ssDNA oligo stock and 100 ng of the CRISPR plasmid with the selected spacer to one side of the wall of the 2‐mm gap width electroporation cuvette.
- Add 100 μl of freshly prepared electrocompetent *P. putida* cells. Proceed to electroporate and recover cells as described in the interference test section.
- Plate appropriate dilutions (10^−2^, 10^−1^ and 10^0^) onto LB agar plus Gm+Sm+Km.

### Verification of target deletion

In order to confirm the complete deletion of the target gene in the transformant colonies, design oligonucleotides that flank that DNA region to perform colony PCR to check the deletion.

- Start by re‐streaking a number of colonies on a fresh LB plate plus Gm+Sm (antibiotic selection for the CRISPR plasmid is not needed in the following steps).
- Perform colony PCR to check the target deletion using the designed primers. To do that, bacterial colonies are picked directly from the agar plate with a sterile toothpick and placed into the PCR tube containing the proper amount of H_2_O. Then, add the Master Mix/Polymerase (following vendor′s instruction) into each PCR tube and proceed with the reaction.
- Select a positive clone and send to sequence the amplified PCR fragment with flanking oligonucleotides (Fig. 2A) to confirm the correctness of the deletion.
- Once the deletion is confirmed, prepare a frozen stock of that clone in LB with 20% (v/v) glycerol to preserve it. Then, if required, proceed to cure the three different plasmids used in the process.

### Curation of plasmids

At the end of the deletion process, we have a bacterial strain that harbours three different plasmids and the last step of this protocol is to eliminate those plasmids from the new engineered strain. A diagram of a general plasmid curation process is represented in Fig. 3B.

The steps that we have to follow in the laboratory are the following:

- Inoculate from the frozen stock a 10 ml test tube containing 3 ml of LB.
- Incubate overnight at 30°C with shaking.
- Prepare a 1/1000 dilution, vortex and transfer 3 μl to a tube with 3 ml of LB.
- Incubate for 6 h at 30°C aerobically.
- Transfer 3 μl of the grown culture (even though no visible growth is observed) to a new tube with 3 ml of fresh LB.
- Incubate overnight at 30°C with shaking.
- Repeat the process from step iii to vi at least 10 times.
- Finally, use the grown liquid culture to streak a LB agar plate to obtain separate colonies.
- Screen several colonies by streaking them onto LB agar plates with and without the proper antibiotics (Sm, Gm and Km).
- Select a colony that is sensitive to all antibiotics used (Sm, Gm and Km) and check again by PCR that is the desired mutated strain (Fig. 3B). Then, prepare a glycerol stock and maintain it at −80°C. If colonies are still resistant to any of the antibiotics, repeat the process from step iii to ix.

## Application examples

With this information in mind, we aimed to test (i) the functionality of the standardized CRISPR plasmid by deleting a target gene (ii) and the possibility of cycling the deletion process by eliminating the curation step of the CRISPR plasmid, speeding the process of making serial deletions into the same strain, increasing the efficiency of the process.

### Deletion of the edd gene of P. putida using ssDNA recombineering and CRISPR/Cas9

The glucose catabolism in *P. putida* occurs almost entirely through the Entner–Doudoroff (ED) pathway and EDEMP cycle (Chavarría *et al*., 2013; Nikel *et al*., 2015). The first enzyme of this pathway is a phosphogluconate dehydratase (EC 4.2.1.12) that catalyses the transformation of 6‐phospho‐D‐gluconate (6PG) to 2‐keto‐3‐deoxy‐6‐phospho‐D‐gluconate (KDPG). This enzyme is encoded by the *edd* (PP_1010) gene. A disruption of this gene prevents the growth of those clones in glycolytic carbon sources but not in gluconeogenic ones or rich media (Nikel *et al*., 2015). For that reason, we selected the *edd* (PP_1010) gene as our deletion target because mutants would show a detectable phenotype (impaired growth on glucose). Moreover, the deleted strain could be interesting *per se* for certain laboratory applications, such as counterselection to discriminate donor and recipient cells in mating‐based experiments between two *Pseudomonas putida* strains.

### Interference test to identify a functional spacer

As recommended in the interference test, it is always a good idea to perform a quick‐and‐dirty experiment to test the functionality of various spacers to choose the most efficient one for the deletion part. For that, we chose two potential spacers that would direct the Cas9 nuclease complex against different regions of the *edd* gene. Thus, we selected two regions of 30 nucleotides that are adjacent to a PAM sequence (Fig. 2 shows the specific example of spacer edd‐1). Then, we cloned the spacers by annealing the oligos cr‐edd‐1‐S with cr‐edd‐1‐AS and cr‐edd‐3‐S with cr‐edd‐3‐AS (Table 2) to yield plasmids pSEVA231‐C‐edd1 and pSEVA231‐C‐edd3 (Table 1). The idea of this simple experiment is that cells containing the Cas9 and the recombinase vector would be transformed with either a CRISPR control plasmid that would not be able to asses a target within the genome and transformed cell thus would be viable (Fig. 3A). On the other hand, cells would be also transformed with a CRISPR plasmid that contain a spacer that would direct the Cas9 complex to a specific genomic target located adjacent to a proper PAM, introducing in that way a DSB that would result in cell death (Fig. 3A).

**Figure 4:**
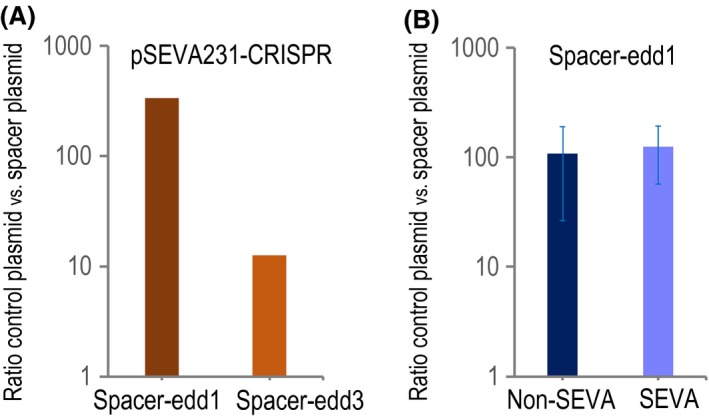
Interference experiments. A. Testing the efficiency of different spacers against the *edd* gene. *P. putida* EM42 harboring pSEVA421‐Cas9tr and pSEVA658‐*ssr* was transformed with either pSEVA231‐CRISPR (control experiment), pSEVA231‐C‐edd1 or pSEVA231‐C‐edd3 and plated on LB+Sm+Gm (to count total viable cells) and on LB+Sm+Gm+Km (to enumerate the number of recombinant clones). For each of the three plasmids the number of recombinant clones was divided by the number of viable cells and the result normalized to a total of 109 cells. Then, the efficiency of the interference was expressed as the ratio of the control plasmid versus the spacer‐harboring one. Since this is a one‐shot experiment, it is important for comparison to transform all plasmids, the control one and all spacers to be tested, within the same electrocompetent batch. The bigger this number is, the better the interference efficiency. In this case, the edd1 spacer showed a better response than edd3. B. Interference comparison of the non‐SEVA versus the SEVA CRISPR plasmids. In this experiment both plasmids contain the same spacer sequence. The average and standard deviation of three biological replicates are shown.

To select the most efficient spacer, we performed an experiment with just one replica. However, in order to properly compare the different plasmids to test, it is important to use the same batch of electrocompetent cells. *P. putida* EM42 (pSEVA658‐*ssr* and pSEVA421‐Cas9tr) was transformed with the following plasmids: pSEVA231‐CRISPR (control), pSEVA231‐C‐edd1 and pSEVA231‐C‐edd3. After 2 h of recovery at 30°C in LB+Sm+Gm, we plated dilutions of transformed cells on LB+Sm+Gm to estimate the number of total viable cells and on LB+Sm+Gm+Km to enumerate the number of transformants. The efficiency of the interference test was calculated, in each case, dividing recombinant clones by viable cells and normalizing that number to 10^9^ cells. To represent the data charted in Fig. 4A, we plotted the ratio of the transformation efficiencies of the control by the CRISPR plasmids. Bigger values represent better interference efficiencies. This is an important parameter since it is going to determine the usability of the spacer. We have observed that values close to 100 allow to perform an efficient counterselection when combined with ssDNA recombineering to delete a gene (Aparicio *et al*., 2017). In this case, plasmid pSEVA231‐C‐edd1 showed a higher interference than pSEVA231‐C‐edd3 (Fig. 4A). So, we selected spacer edd1 for future experiments and discarded the spacer edd3.

### Assaying the functionality of the new pSEVA2316 plasmid

Our next objective was to evaluate the functionality of the new constructed CRISPR module. Therefore, we cloned the most efficient spacer, edd1, in plasmid pSEVA2316 to render pSEVA2316‐edd1 (Table 1). Then, we proceed to compare the efficiency of the SEVA rule violating, pSEVA231‐C‐edd1, and the SEVA plasmid, pSEVA2316‐edd1 (Fig. 4B). In this case, we performed three biological replicates and represented the average and standard deviation. As shown in Fig. 4B, both plasmids display similar interference values (*p* value of 0.79; unpaired t test with a significance threshold set at 0.05), demonstrating that the SEVA version of the CRISPR plasmid shows the same functionality. On these terms, we select the SEVA version for the following experiments.

### Target edd gene deletion

**Figure 5:**
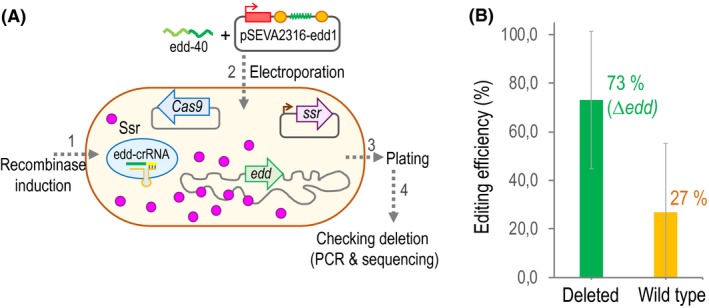
Deletion of the *edd* gene with ssDNA recombineering and CRISPR/Cas9 counterselection. A. An illustration of the steps required to do the protocol. The first step is to load the cell with the Ssr recombinase to protect the ssDNA oligo that would be transformed alongside the CRISPR plasmid (step 2). The step 3 is to recover cells after the electro‐shock and to plate dilutions onto appropriate media. Finally, the last step of the process is to check the deletion by PCR and further corroboration by DNA sequencing. B. Editing efficiency obtained for the 1kb fragment deletion of the *edd* gene. The EM42 strain with pSEVA421‐Cas9tr and pSEVA658‐*ssr* was transformed together with edd‐40 oligo and the pSEVA2316‐edd1 plasmid. Then, the editing efficiency was calculated as the percentage of mutated clones versus non‐modified sequences. The chart represents the average and standard deviation of two biological replicates.

Once selected an efficient spacer and tested the functionality of the pSEVA2316‐edd1, the next step was to proceed with the ssDNA recombineering to delete a 1 kb DNA fragment of the *edd* gene and the counterselection exhorted by the CRISPR/Cas9 system as a proof of concept. A scheme of the whole process is depicted in Fig. 5A. Briefly, cells loaded with the Ssr recombinase are co‐transformed with 1 μl of the ∼100 μM mutagenic oligonucleotide edd‐40 (Table 2) and 100 ng of the CRISPR plasmid that has the appropriate spacer (pSEVA2316‐edd1; Table 1) to kill unmodified cells. Transformed cells were recovered for 2 h at 30°C in LB+Sm+Gm and plated on selective LB media containing Sm+Gm+Km to recover clones with the three plasmids (Fig. 5A). Two different morphologies, big and small colonies, were cleary visible in the plate. Then, individual clones of each morphology were subjected to colony PCR, using oligos edd‐check‐F and edd‐check‐R (Table 2), to verify whether they have the desired mutation or not. It turned out that all small colonies were deleted strains while the big ones corresponded to wild‐type cells. In order to calculate the editing efficiency, we considered all colonies, big and small, that appeared on the selective plate. We repeated the experiment twice and plotted the average with the standard deviation. The observed deletion efficiency of the *edd* gene was 73% while 27% of the colonies remained wild type (Fig. 5B). Moreover, we selected a total of six mutated clones and the genomic region of *edd* was sequenced to validate the accuracy of the deletion. All clones showed the expected sequence in the deleted region. Therefore, this approach establishes a powerful and reliable genome engineering tool, allowing to obtain a deleted strain within a few days.

### Cycling the deletion process

**Figure 6:**
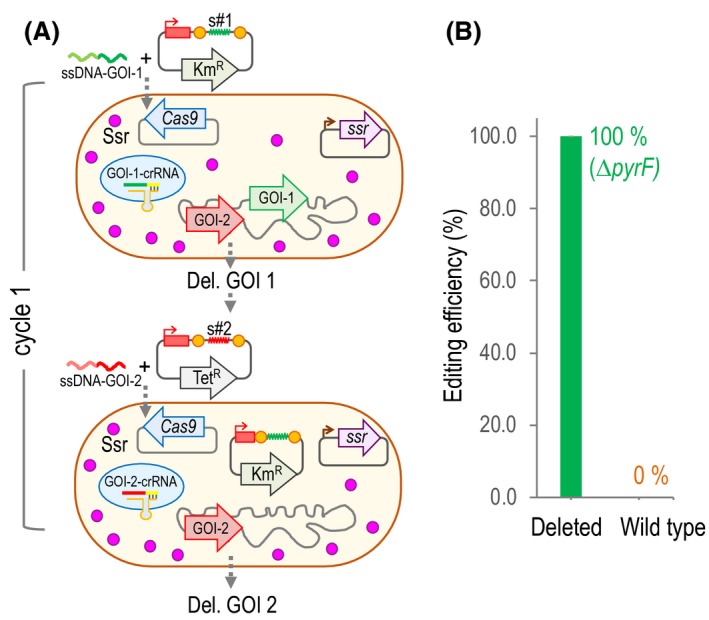
Steps of the cycled deletion process**.** A. A schematic representation of the steps required to cycle a deletion experiment. First, a strain harbouring pSEVA421‐Cas9tr and pSEVA658‐*ssr* and fully loaded with the Ssr recombinase is transformed with the ssDNA‐GOI‐1 (Gene Of Interest #1) and CRISPR‐spacer‐#1 plasmid (Km^R^) to perform the deletion of the GOI‐1. Once the deletion of the GOI‐1 is confirmed, the strain is directly transformed with the ssDNA‐GOI‐2 and with a CRISPR‐spacer‐#2 plasmid (Tet^R^) to perform the second targeted deletion. These two deletions comprise one cycle. Then, after selecting a Tet^R^‐Km^S^ ∆GO‐1∆GOI‐2 clone, ideally a third deletion could be performed directly with a Km^R^ CRISPR‐spacer‐#3 plasmid. In theory, this process can be cycled indefinitely to perform an X number of deletions within the same background. B. Editing efficiency of the *pyrF* deletion in an EM42∆*edd* strain harbouring the pSEVA421‐Cas9tr, pSEVA658‐*ssr* and pSEVA2316‐edd1 plasmids. That strain was co‐transformed with the pyrF‐B‐np oligo and the pSEVA5316‐pyrF1 plasmid. The average and standard deviation of two biological replicates are plotted.

In certain cases, it is necessary to introduce more than one deletion in the same strain. We wanted to test the possibility of cycling the deletion process without curing the CRISPR plasmid. Meaning by that, to directly electroporate the strain with a deletion in the Gene Of Interest‐1 (GOI‐1) with both mutagenic oligo#2 and CRISPR plasmid‐spacer #2. Since both CRISPR plasmids share the same replicon (pBBR1), it is important to use two plasmids with different selection markers (Km^R^ and Tet^R^ in this example). Once confirmed the second deletion event (strain GOI‐2), a third round could potentially be introduced by re‐using again the Km^R^ CRISPR plasmid‐bearing spacer #3. Then, a fourth round could be done using the Tet^R^ CRISPR plasmid‐bearing spacer #4. An illustration of the process is depicted in Fig. 6A. So as to test whether this cycling process is doable, we planned to perform two deletions within the same strain. The selected targets were the previously deleted *edd* and the *pyrF* gene (PP_1815), whose disruption generates a strain auxothoph for uracil (Aparicio *et al*., 2016). The spacer pyrF1 was designed and tested in a previous work into the pSEVA231‐CRISPR plasmid (Aparicio *et al*., 2017). To perform this cycled experiment, we cloned the pyrF1 spacer into a Tet^R^ CRISPR plasmid (pSEVA5316) to obtain pSEVA5316‐pyrF1 plasmid (Table 1).

Once obtained a *P. putida* EM42 with an *edd* deletion (as described above), we transformed that strain with the pyrF‐B‐np oligo (Table 2) and the pSEVA5316‐pyrF1 plasmid (Table 1) and plated dilutions on LB+Sm+Gm+Tet+Ura. The experiment was done twice and all colonies tested (*n* = 20) corresponded to *pyrF* deleted strains, accounting a 100% efficiency (Fig. 6B). Of those, we selected 6 mutated clones and confirmed the deletion by sequencing the appropriate genomic region. All clones had the expected sequence throughout the deletion boundaries. Then, we tested whether the second CRISPR plasmid (Tet^R^) displaced the first one (Km^R^) or both were able to coexist in bacterial cells. To do that, we selected a number of clones with a double deletion that were Sm^R^Gm^R^Tet^R^ and re‐streaked those on similar plates supplemented with Km. Of a total of 35 clones, ~ 83% were Km^R^, denoting that were able to maintain both CRISPR plasmids, while ~ 17% were Km^S^, indicating that they lost the first CRISPR plasmid and only kept the second one. The plasmid displacement occurred at a doable frequency, allowing to establish a serial deletion protocol that might speed up multiple deletions.

**Figure 7:**
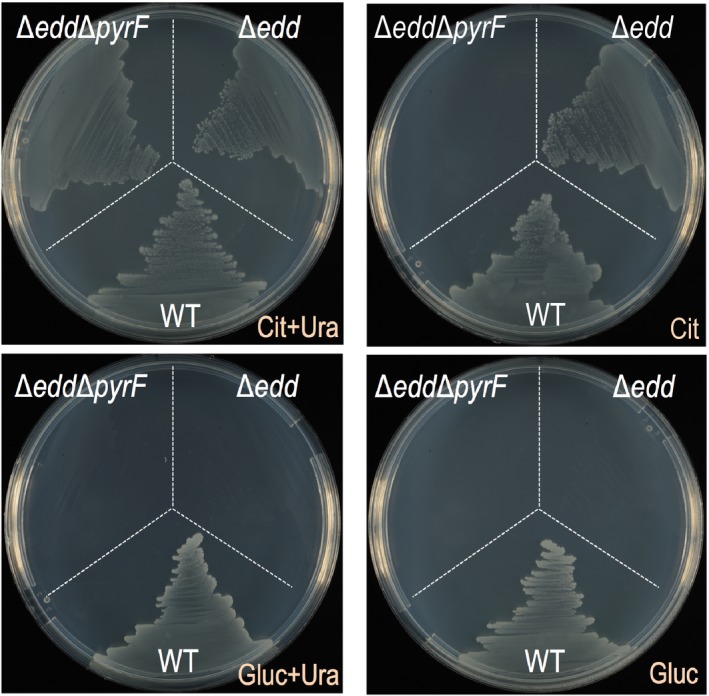
Phenotypic characterization of the *P. putida* mutants Δ*edd and* Δ*edd* Δ*pyrF*. The single and the double deletion mutants together with the parental EM42 (WT) strain were streaked out on M9 minimal agar plates supplemented with: citrate and uracil, citrate, glucose plus uracil and glucose.

Finally, after having the desired ∆e*dd* ∆*pyrF* strain, we proceeded to cure the three working plasmids (Fig. 3B). To start this process, we selected a double mutant clone that was Km^S^ and inoculated an LB tube without antibiotics, performing five consecutive passes of curation. After those, we checked 20 individual colonies and confirmed that they lost the pSEVA421‐Cas9tr (Sm^R^) and pSEVA5316‐pyrF1 (Tet^R^) but all of them still had the high/medium copy plasmid pSEVA658‐*ssr* (Gm^R^). Then, we started the process again and continued it for an extra 10 more cycles. After that, we analysed a total of 150 colonies and 98% did lose the pSEVA658‐*ssr* plasmid. Doing this protocol, we constructed a *P. putida* strain (EM42∆e*dd*∆*pyrF*) that is unable to grow on glucose as C‐source and also shows auxotrophy for uracil. To phenotypically confirm that, we streaked the wild type, the ∆e*dd* mutant and double mutant (∆e*dd*∆*pyrF*) on four different media (Fig. 7). On M9 + cit+uracil, as expected, the three strains were able to grow. Then, on M9 + citrate only the wild type and ∆e*dd* mutants grew. Similarly, when using glucose as C‐source, neither of the *edd* mutants were able to grow.

## Outlook

**Figure 8:**
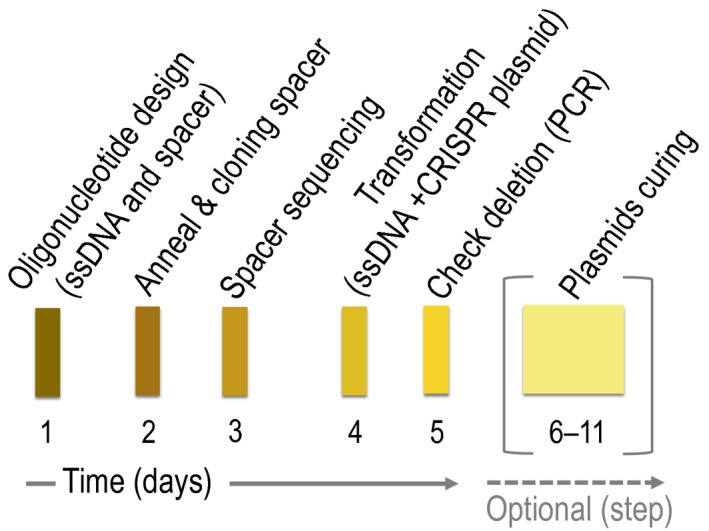
Workflow of the use of ssDNA recombineering combined with CRISPR/Cas9 for genome editing. This diagram represents the key steps of this protocol. The first thing to do is to design the ssDNA for recombineering and selection of spacer/s within the region to be deleted. Order those oligos with your favourite supplier. Upon receiving those oligos, anneal the S and AS spacer oligos and clone those into the CRISPR plasmid. On the same day, transform your favourite *E. coli* laboratory strain. The next day, select a few clones and grow them for ∼ 6 h to isolate plasmids. Then, sequence them to confirm the presence of the proper spacer. After that, just electroporate the ssDNA and CRISPR plasmid into the studied strain and plate onto appropriate medium. Finally, check the presence of the correct deletion by PCR. This makes the whole process doable within a week time. The last and optional step of the process involves the curing of the working plasmids that it is easily obtained by just growing the deleted clone in LB without antibiotics for a number of generations.

In this article, we examined the combined use of ssDNA recombineering with CRISPR/Cas9 as the counterselection technique to re‐write bacterial genomes. The present protocol allows to do genome engineering in *P. putida* not only in a shorter time than homologous recombination‐based approaches, but it also demands less laboratory work than other procedures (Martinez‐Garcia and de Lorenzo, 2011, 2012). One simple cloning step for the construction of the CRISPR plasmid containing the spacer is required. Here, we extensively described all the steps needed to do a genome modification using *P. putida* as the example organism. Fig. 8 summarizes all the steps of the process in a simple workflow. However, this protocol could be adjusted easily to work in any *Pseudomonas* species. To facilitate that, we also SEVArized the CRISPR array, eliminating the PshAI restriction site present within the leader region, to render the new cargo assigned with the code #16. We included that cargo into the pSEVA231 and pSEVA531 plasmids to yield the pSEVA2316 and pSEVA5316 vectors that would be included in the SEVA database. Since these plasmids are modular now, the antibiotic selection marker of these plasmids could be easily swapped by the use of SwaI and PshAI restriction enzymes with any other cassette of the collection at user′s will. The use of these two CRISPR plasmids together with the pSEVA421‐Cas9tr and pSEVA658‐*ssr* allows to cycle the deletion process without the need to cure the previously used CRISPR plasmid. This cycled process is especially indicated for cases where one needs to do several genome modifications within the same strain. This protocol could be further optimized, for instance by reducing the number of working plasmids, but at the end of the day the general idea and the steps of this procedure would be similar.

## Conflict of interest

None declared.
